# Gene expression in primary and metastatic kidney cancer to discover drivers of metastasis and targets for drug development

**DOI:** 10.1186/2051-1426-3-S2-P215

**Published:** 2015-11-04

**Authors:** Amy Zhao, Arjun Guru, Sonpavde Guru, Eddy Yang, Yufeng Li

**Affiliations:** 1 University of Alabama at Birmingham, Birmingham, AL, USA

## Background

An estimated 63,920 cases of kidney cancer will occur in the USA in 2015, and about 13,860 deaths will occur. The incidences of kidney cancer has steadily increased by 2-4% each year. Currently high dose IL (Interleukin)-2, VEGF (vascular endothelial growth factor) inhibitors, and mTOR (mammalian target of rapamycin) inhibitors are often used to treat metastatic kidney cancer, but all of the above treatments lead to a median survival between 1-2 years.

Therefore, there is a large role for discovering new drugs against new molecules that drive cancer growth. Although the understanding of biology leading to better treatments will likely require analysis of metastatic tumor tissue, most studies have only analyzed the primary tumor. We studied both primary and metastatic tumors from FFPE tissue to identify new kinase genes expressed in metastatic tumors compared to primary kidney tumors. These data may enable the design of new drugs, since it is relatively easier to design kinase inhibitors.

## Methods

A total of 54 samples of primary tumor, adjacent normal kidney, and metastatic tumor from 18 patients were available. RNA from FFPE tissue samples was analyzed using Nanostring technology for expression of 519 kinase genes. This technology is robust for studying gene expression, even from FFPE tissue. The data was analyzed using the nCounter platform (NanoString Technologies, Seattle, WA). Data quality check and normalization was performed prior to analysis. Over-expression was defined as at least 2-fold elevation compared to reference genes. Descriptive analysis and unsupervised cluster analysis were applied. This study has approval of Institutional Review Board.

## Results

There is a cluster of kinase genes over expressed in metastasis, but not in normal and primary tumor. ROS1, EPHA3, PLK1 and CDK1 were among the top 10 genes over-expressed in metastases compared to either primary tumor or normal kidney. All of the top kinases genes over-expressed in metastases in our study are known to drive growth, survival and metastasis of tumor cells. There are 8 unique genes over-expressed only in metastasis compared to primary tumor (CDK7, ICK, KDR, LTK, OBSCN, PRKCH, RIPK3 and SGK110). Some genes were under-expressed in tumors compared to normal kidney, suggesting that some kinase genes could protect against tumor growth.

## Conclusions

We identified genes only overexpressed in metastatic kidney tumor tissue, which may represent drivers of metastasis and targets for new drugs. Confirmation of findings is required before clinical trials of new drugs.

